# *N*-Butyl-4-chloro­benzamide

**DOI:** 10.1107/S1600536808036313

**Published:** 2008-11-13

**Authors:** Aamer Saeed, Rasheed Ahmad Khera, Naeem Abbas, Jim Simpson, Roderick G. Stanley

**Affiliations:** a Department of Chemistry, Quaid-i-Azam University, Islamabad 45320, Pakistan; b Department of Chemistry, University of Otago, PO Box 56, Dunedin, New Zealand

## Abstract

In the title benzamide derivative, C_11_H_14_ClNO, the chloro­benzene and butyl­amine groups are each planar, with mean deviations from the planes of 0.013 and 0.030 Å, respectively, and a dihedral angle of 2.54 (9)° between the two planes. In the crystal structure, N—H⋯O hydrogen bonds link mol­ecules in rows along *a*. Short inter­molecular Cl⋯Cl inter­actions [3.4225 (5) Å] link these rows into sheets in the *ac* plane. Additional weak C—H⋯O and C—H⋯π inter­actions generate a three-dimensional network.

## Related literature

For details of the biological activity of benzanilides, see: Olsson *et al.*, (2002 ▶); Lindgren *et al.* (2001 ▶); Calderone *et al.* (2006 ▶). For the use of benzamides in organic synthesis, see: Reinaud *et al.* (1991 ▶); Zhichkin *et al.* (2007 ▶); Beccalli *et al.* (2005 ▶); For the fluorescence properties of benzanilides, see: Lewis & Long (1998 ▶). For related structures see: Saeed *et al.* (2008 ▶); Hempel *et al.* (2005 ▶). For reference structural data, see: Allen *et al.* (1987 ▶). For related literature, see: Vega-Noverola *et al.* (1989 ▶); Yoo *et al.* (2005 ▶).
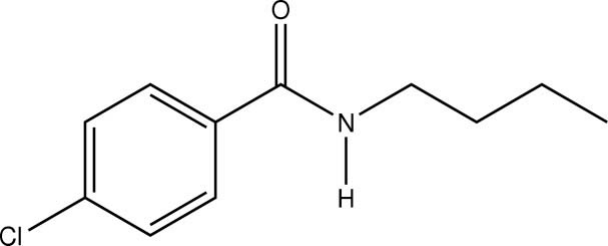


## Experimental

### 

#### Crystal data

C_11_H_14_ClNO
*M* *_r_* = 211.68
Triclinic, 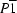

*a* = 5.1702 (4) Å
*b* = 7.8979 (5) Å
*c* = 13.2978 (9) Å
α = 89.275 (3)°
β = 84.863 (4)°
γ = 77.165 (4)°
*V* = 527.29 (6) Å^3^
*Z* = 2
Mo *K*α radiation
μ = 0.33 mm^−1^
*T* = 81 (2) K
0.42 × 0.30 × 0.08 mm

#### Data collection

Bruker APEXII CCD area-detector diffractometer
Absorption correction: multi-scan (*SADABS*; Bruker, 2006 ▶) *T*_min_ = 0.820, *T*_max_ = 0.974
6632 measured reflections
3445 independent reflections
3050 reflections with *I* > 2σ(*I*)
*R*_int_ = 0.017

#### Refinement

*R*[*F* ^2^ > 2σ(*F* ^2^)] = 0.033
*wR*(*F* ^2^) = 0.090
*S* = 1.04
3445 reflections
132 parameters
H atoms treated by a mixture of independent and constrained refinement
Δρ_max_ = 0.43 e Å^−3^
Δρ_min_ = −0.22 e Å^−3^

### 

Data collection: *APEX2* (Bruker, 2006 ▶); cell refinement: *APEX2* and *SAINT* (Bruker, 2006 ▶); data reduction: *SAINT*; program(s) used to solve structure: *SHELXS97* (Sheldrick, 2008 ▶); program(s) used to refine structure: *SHELXL97* (Sheldrick, 2008 ▶) and *TITAN2000* (Hunter & Simpson, 1999 ▶); molecular graphics: *ORTEP-3* (Farrugia, 1997 ▶) and *Mercury* (Macrae *et al.*, 2006 ▶); software used to prepare material for publication: *SHELXL97*, *enCIFer* (Allen *et al.*, 2004 ▶), *PLATON* (Spek, 2003 ▶) and *publCIF* (Westrip, 2008 ▶).

## Supplementary Material

Crystal structure: contains datablocks global, I. DOI: 10.1107/S1600536808036313/sg2272sup1.cif

Structure factors: contains datablocks I. DOI: 10.1107/S1600536808036313/sg2272Isup2.hkl

Additional supplementary materials:  crystallographic information; 3D view; checkCIF report

## Figures and Tables

**Table 1:** Hydrogen-bond geometry (Å, °)

*D*—H⋯*A*	*D*—H	H⋯*A*	*D*⋯*A*	*D*—H⋯*A*
N1—H*N*1⋯O1^i^	0.831 (15)	2.203 (15)	3.0164 (10)	166.3 (13)
C3—H3⋯O1^ii^	0.95	2.66	3.3146 (11)	127
C8—H8*A*⋯*Cg*1^iii^	0.99	2.84	3.697 (16)	145

Symmetry codes: (i) 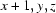; (ii) 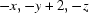; (iii) 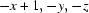. *Cg*1 is the centroid of the C2–C7 benzene ring.

**Fig. 1.**
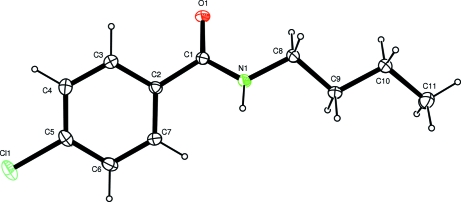
The structure of I showing the atom numbering with displacement ellipsoids drawn at the 50% probability level.

**Fig. 2.**
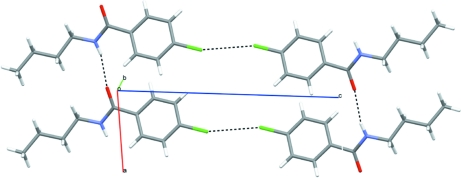
Sheets of molecules of I formed in the ac plane by N—H···O hydrogen bonds and Cl···Cl interactions.

**Fig. 3.**
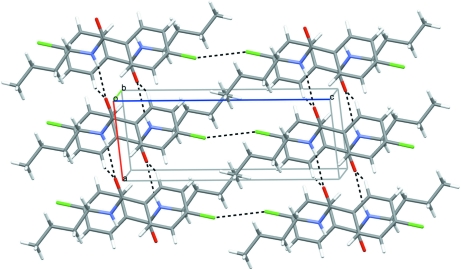
Crystal packing of I viewed down the b axis.

## supplementary crystallographic information

### Comment

The benzanilide core is present in compounds with such a wide range of biological activities that it has been called a privileged structure. N-substituted benzamides are well known anticancer compounds and the mechanism of action for N-substituted benzamide-induced apoptosis has been studied, using declopramide as a lead compound (Olsson *et al.*, 2002). N-substituted benzamides inhibit the activity of nuclear factor- B and nuclear factor of activated T cells activity while inducing activator protein 1 activity in T lymphocytes (Lindgren *et al.*, 2001). Various N-substituted benzamides exhibit potent antiemetic activity (Vega-noverola *et al.*, 1989), while heterocyclic analogs of benzanilide derivatives are potassium channel activators (Calderone *et al.*, 2006). o-Aryloxylation of N-substituted benzamides induced by the copper(II)/trimethylamine N-oxide system has been studied (Reinaud *et al.*, 1991). N-Alkylated 2-nitrobenzamides are intermediates in the synthesis of dibenzo[b,e][1,4]diazepines (Zhichkin *et al.*, 2007) and N-Acyl-2-nitrobenzamides are precursors of 2,3-disubstitued 3H-quinazoline-4-ones (Beccalli *et al.*, 2005). A one-pot conversion of 2-nitro-n-arylbenzamides to 2,3-dihydro-1H-quinazoline-4-ones has also been reported (Yoo *et al.*, 2005). The anomalous dual fluorescence of benzanilides has been assigned to the two lowest benzanilide singlet states (Lewis & Long, 1998)

As part of our work on the structure of benzanildes and related compounds, we report here the structure of the title benzamide derivative, I, Fig. 1. The C1···C7/Cl system is planar with a maximum deviation of 0.0161 (7) Å from the least squares plane. The carbonyl oxygen atom O1 is displaced by 0.6102 (10) Å from this plane. The butylamine N1/C8···C11 fragment is also planar, maximum deviation 0.0365 (7) Å for C9. The dihedral angle between these two planes is 2.54 (9) °. Bond distances within the molecule are normal (Allen *et al.*, 1987) and similar to those found in the structures of related 4-chlorobenzamide derivatives (Saeed *et al.*, 2008, Hempel *et al.*, 2005).

In the crystal structure N1—HN1···O1 hydrogen bonds, Table 1, link molecules into rows along *a*. Cl1···Cl1 interactions at 3.4225 (5) Å bridge these rows to form sheets in the ac plane, Fig. 2. The sheets are interconnected by weak C3—H3···O1 hydrogen bonds and C8—H8···π interactions involving the C2···C7 benzene ring to generate a three dimensional network, Fig. 3.

### Experimental

2-Fluorobenzoyl chloride (1 mmol) in CHCl_3_ was treated with cyclohexyl amine (3.5 mmol) under a nitrogen atmosphere at reflux for 5 h. Upon cooling, the reaction mixture was diluted with CHCl_3_ and washed consecutively with 1 *M* aq HCl and saturated aq NaHCO_3_. The organic layer was dried over anhydrous sodium sulfate and concentrated under reduced pressure. Crystallization of the residue in ethanol afforded the title compound (79 %) as white needles: Anal. calcd. for C_11_H_14_ClNO: C 62.41, H 6.67, N 6.62%; found: C 62.34, H 7.16, N 6.57%.

### Refinement

The H atom bound to N1 was located in a difference electron density map and refined freely with an isotropic displacement parameter. All other H-atoms were refined using a riding model with d(C—H) = 0.95 Å, *U*_iso_= 1.2*U*_eq_ (C) for aromatic, 0.99Å, U_iso_ = 1.2U_eq_ (C) for CH_2_, and 0.98 Å, *U*_iso_ = 1.5*U*_eq_ (C) for CH_3_ H atoms.

### Crystal data

**Table:**

C_11_H_14_ClNO	*Z* = 2
*M**_r_* = 211.68	*F*_000_ = 224
Triclinic, *P*1	*D*_x_ = 1.333 Mg m^−^^3^
Hall symbol: -P 1	Mo *K*α radiation λ = 0.71073 Å
*a* = 5.1702 (4) Å	Cell parameters from 3494 reflections
*b* = 7.8979 (5) Å	θ = 5.3–66.2º
*c* = 13.2978 (9) Å	µ = 0.33 mm^−^^1^
α = 89.275 (3)º	*T* = 81 (2) K
β = 84.863 (4)º	Irregular fragment, colourless
γ = 77.165 (4)º	0.42 × 0.30 × 0.08 mm
*V* = 527.29 (6) Å^3^	

### Data collection

**Table:**

Bruker APEXII CCD area-detector diffractometer	3445 independent reflections
Radiation source: fine-focus sealed tube	3050 reflections with *I* > 2σ(*I*)
Monochromator: graphite	*R*_int_ = 0.017
*T* = 81(2) K	θ_max_ = 33.1º
ω scans	θ_min_ = 3.1º
Absorption correction: multi-scan(SADABS; Bruker, 2006)	*h* = −7→6
*T*_min_ = 0.820, *T*_max_ = 0.974	*k* = −11→11
6632 measured reflections	*l* = −20→20

### Refinement

**Table:**

Refinement on *F*^2^	Secondary atom site location: difference Fourier map
Least-squares matrix: full	Hydrogen site location: inferred from neighbouring sites
*R*[*F*^2^ > 2σ(*F*^2^)] = 0.033	H atoms treated by a mixture of independent and constrained refinement
*wR*(*F*^2^) = 0.090	*w* = 1/[σ^2^(*F*_o_^2^) + (0.0471*P*)^2^ + 0.125*P*] where *P* = (*F*_o_^2^ + 2*F*_c_^2^)/3
*S* = 1.04	(Δ/σ)_max_ = 0.002
3445 reflections	Δρ_max_ = 0.43 e Å^−^^3^
132 parameters	Δρ_min_ = −0.22 e Å^−^^3^
Primary atom site location: structure-invariant direct methods	Extinction correction: none

### Special details

**Table:**

Geometry. All esds (except the esd in the dihedral angle between two l.s. planes) are estimated using the full covariance matrix. The cell esds are taken into account individually in the estimation of esds in distances, angles and torsion angles; correlations between esds in cell parameters are only used when they are defined by crystal symmetry. An approximate (isotropic) treatment of cell esds is used for estimating esds involving l.s. planes.
Refinement. Refinement of F^2^ against ALL reflections. The weighted R-factor wR and goodness of fit S are based on F^2^, conventional R-factors R are based on F, with F set to zero for negative F^2^. The threshold expression of F^2^ > 2sigma(F^2^) is used only for calculating R-factors(gt) etc. and is not relevant to the choice of reflections for refinement. R-factors based on F^2^ are statistically about twice as large as those based on F, and R- factors based on ALL data will be even larger.

### Fractional atomic coordinates and isotropic or equivalent isotropic displacement parameters (Å^2^)

**Table:**

	*x*	*y*	*z*	*U*_iso_*/*U*_eq_	
N1	0.51637 (16)	0.82511 (10)	−0.10915 (6)	0.01587 (15)	
HN1	0.662 (3)	0.8260 (18)	−0.0876 (11)	0.026 (3)*	
C1	0.31476 (17)	0.79695 (10)	−0.04475 (6)	0.01281 (15)	
O1	0.08420 (13)	0.81551 (8)	−0.06861 (5)	0.01616 (14)	
C2	0.38267 (17)	0.73835 (10)	0.05917 (6)	0.01257 (15)	
C3	0.18549 (19)	0.78474 (11)	0.13885 (7)	0.01611 (17)	
H3	0.0169	0.8545	0.1262	0.019*	
C4	0.2331 (2)	0.73001 (12)	0.23650 (7)	0.01847 (18)	
H4	0.0995	0.7631	0.2908	0.022*	
C5	0.4800 (2)	0.62579 (11)	0.25329 (7)	0.01667 (17)	
Cl1	0.54360 (5)	0.55693 (3)	0.375283 (17)	0.02530 (8)	
C6	0.67765 (19)	0.57568 (12)	0.17528 (7)	0.01740 (17)	
H6	0.8439	0.5027	0.1879	0.021*	
C7	0.62886 (18)	0.63406 (11)	0.07801 (7)	0.01532 (16)	
H7	0.7642	0.6026	0.0241	0.018*	
C8	0.47533 (19)	0.87979 (13)	−0.21289 (7)	0.01730 (17)	
H8A	0.4281	1.0082	−0.2152	0.021*	
H8B	0.3242	0.8363	−0.2352	0.021*	
C9	0.72100 (18)	0.81319 (12)	−0.28494 (7)	0.01526 (16)	
H9A	0.7664	0.6847	−0.2840	0.018*	
H9B	0.8733	0.8549	−0.2623	0.018*	
C10	0.67469 (19)	0.87506 (12)	−0.39261 (7)	0.01705 (17)	
H10A	0.5112	0.8435	−0.4123	0.020*	
H10B	0.6458	1.0032	−0.3943	0.020*	
C11	0.9070 (2)	0.79692 (14)	−0.46899 (8)	0.02213 (19)	
H11A	1.0696	0.8277	−0.4500	0.033*	
H11B	0.8691	0.8425	−0.5363	0.033*	
H11C	0.9317	0.6703	−0.4698	0.033*	

### Atomic displacement parameters (Å^2^)

**Table:**

	*U*^11^	*U*^22^	*U*^33^	*U*^12^	*U*^13^	*U*^23^
N1	0.0106 (3)	0.0251 (4)	0.0134 (3)	−0.0064 (3)	−0.0033 (3)	0.0048 (3)
C1	0.0120 (4)	0.0133 (3)	0.0131 (4)	−0.0024 (3)	−0.0018 (3)	0.0006 (3)
O1	0.0097 (3)	0.0217 (3)	0.0170 (3)	−0.0028 (2)	−0.0029 (2)	0.0024 (2)
C2	0.0115 (4)	0.0138 (3)	0.0131 (4)	−0.0038 (3)	−0.0025 (3)	0.0013 (3)
C3	0.0132 (4)	0.0188 (4)	0.0154 (4)	−0.0017 (3)	−0.0005 (3)	0.0009 (3)
C4	0.0185 (4)	0.0224 (4)	0.0140 (4)	−0.0043 (3)	0.0006 (3)	0.0006 (3)
C5	0.0213 (4)	0.0170 (4)	0.0138 (4)	−0.0074 (3)	−0.0055 (3)	0.0036 (3)
Cl1	0.03326 (15)	0.02986 (13)	0.01517 (12)	−0.00986 (10)	−0.00874 (9)	0.00705 (8)
C6	0.0160 (4)	0.0180 (4)	0.0184 (4)	−0.0030 (3)	−0.0058 (3)	0.0038 (3)
C7	0.0117 (4)	0.0177 (4)	0.0159 (4)	−0.0019 (3)	−0.0015 (3)	0.0016 (3)
C8	0.0121 (4)	0.0261 (4)	0.0137 (4)	−0.0040 (3)	−0.0022 (3)	0.0059 (3)
C9	0.0115 (4)	0.0202 (4)	0.0143 (4)	−0.0034 (3)	−0.0028 (3)	0.0018 (3)
C10	0.0138 (4)	0.0230 (4)	0.0139 (4)	−0.0032 (3)	−0.0020 (3)	0.0029 (3)
C11	0.0185 (5)	0.0300 (5)	0.0170 (4)	−0.0043 (4)	0.0006 (3)	−0.0014 (3)

### Geometric parameters (Å, °)

**Table:**

N1—C1	1.3446 (12)	C6—H6	0.9500
N1—C8	1.4598 (11)	C7—H7	0.9500
N1—HN1	0.831 (15)	C8—C9	1.5185 (13)
C1—O1	1.2378 (11)	C8—H8A	0.9900
C1—C2	1.4984 (12)	C8—H8B	0.9900
C2—C3	1.3952 (12)	C9—C10	1.5290 (12)
C2—C7	1.3955 (12)	C9—H9A	0.9900
C3—C4	1.3891 (13)	C9—H9B	0.9900
C3—H3	0.9500	C10—C11	1.5242 (13)
C4—C5	1.3918 (13)	C10—H10A	0.9900
C4—H4	0.9500	C10—H10B	0.9900
C5—C6	1.3848 (14)	C11—H11A	0.9800
C5—Cl1	1.7405 (9)	C11—H11B	0.9800
C6—C7	1.3931 (12)	C11—H11C	0.9800
			
C1—N1—C8	121.48 (8)	N1—C8—C9	112.18 (7)
C1—N1—HN1	119.5 (10)	N1—C8—H8A	109.2
C8—N1—HN1	118.4 (10)	C9—C8—H8A	109.2
O1—C1—N1	122.89 (8)	N1—C8—H8B	109.2
O1—C1—C2	120.60 (8)	C9—C8—H8B	109.2
N1—C1—C2	116.51 (8)	H8A—C8—H8B	107.9
C3—C2—C7	119.40 (8)	C8—C9—C10	111.15 (7)
C3—C2—C1	117.87 (8)	C8—C9—H9A	109.4
C7—C2—C1	122.67 (8)	C10—C9—H9A	109.4
C4—C3—C2	120.71 (8)	C8—C9—H9B	109.4
C4—C3—H3	119.6	C10—C9—H9B	109.4
C2—C3—H3	119.6	H9A—C9—H9B	108.0
C3—C4—C5	118.78 (9)	C11—C10—C9	112.75 (8)
C3—C4—H4	120.6	C11—C10—H10A	109.0
C5—C4—H4	120.6	C9—C10—H10A	109.0
C6—C5—C4	121.65 (8)	C11—C10—H10B	109.0
C6—C5—Cl1	118.96 (7)	C9—C10—H10B	109.0
C4—C5—Cl1	119.39 (7)	H10A—C10—H10B	107.8
C5—C6—C7	118.95 (8)	C10—C11—H11A	109.5
C5—C6—H6	120.5	C10—C11—H11B	109.5
C7—C6—H6	120.5	H11A—C11—H11B	109.5
C6—C7—C2	120.49 (9)	C10—C11—H11C	109.5
C6—C7—H7	119.8	H11A—C11—H11C	109.5
C2—C7—H7	119.8	H11B—C11—H11C	109.5
			
C8—N1—C1—O1	−0.14 (13)	C3—C4—C5—Cl1	179.68 (7)
C8—N1—C1—C2	179.00 (8)	C4—C5—C6—C7	1.22 (14)
O1—C1—C2—C3	−30.72 (12)	Cl1—C5—C6—C7	−178.56 (7)
N1—C1—C2—C3	150.11 (8)	C5—C6—C7—C2	−1.35 (13)
O1—C1—C2—C7	146.41 (9)	C3—C2—C7—C6	0.37 (13)
N1—C1—C2—C7	−32.76 (12)	C1—C2—C7—C6	−176.72 (8)
C7—C2—C3—C4	0.79 (13)	C1—N1—C8—C9	−149.00 (8)
C1—C2—C3—C4	178.01 (8)	N1—C8—C9—C10	−178.91 (7)
C2—C3—C4—C5	−0.92 (14)	C8—C9—C10—C11	−174.52 (8)
C3—C4—C5—C6	−0.10 (14)		

### Hydrogen-bond geometry (Å, °)

**Table:**

*D*—H···*A*	*D*—H	H···*A*	*D*···*A*	*D*—H···*A*
N1—HN1···O1^i^	0.831 (15)	2.203 (15)	3.0164 (10)	166.3 (13)
C3—H3···O1^ii^	0.95	2.66	3.3146 (11)	127
C8—H8A···Cg1^iii^	0.99	2.84	3.697 (16)	145

Symmetry codes: (i) *x*+1, *y*, *z*; (ii) −*x*, −*y*+2, −*z*; (iii) −*x*+1, −*y*, −*z*.
